# Linguistic translation and validation of the Arabic version of International Female Coital Incontinence Questionnaire (IFCI-Q)

**DOI:** 10.1080/2090598X.2023.2242162

**Published:** 2023-08-01

**Authors:** Wally Mahfouz, Ahmed Moussa, Maurizio Serati, Matteo Balzarro, Emanuele Rubilotta, Marilena Gubbiotti

**Affiliations:** a Urology Department, Alexandria University, Alexandria, Egypt; b Department of Obstetrics and Gynecology, University of Insubria, Varese, Italy; c Urology Department, A.O.U.I. Verona University, Italy; d Urology Department, San Donato Hospital, Arezzo, Italy

**Keywords:** Coital urinary incontinence, IFCI-Q, linguistic translation validated questionnaire

## Abstract

**Objectives:**

Aim of the study was to translate the International Female Coital Incontinence Questionnaire (IFCI-Q) into Arabic (Egyptian) and validate it into among Egyptian population complaining of coital urinary incontinence (CI).

**Methods:**

Original questionnaire has been translated and back-translated by an expert panel, to produce the Arabic version. A pilot study was performed to make sure the questionnaire was understandable. Sixty patients included in the study were divided into two groups: Group A comprised patients with CI, and Group B comprised females who attended the urology clinic for other complaints, without CI. Reliability of the Arabic IFCI-Q was evaluated for internal consistency using Cronbach alpha coefficient. Test–retest reliability was determined using the Weighted Cohen’s k-test. Discrimination validity was evaluated by comparing scores of patients with those of healthy females not complaining of CI using Mann–Whitney test.

**Results:**

83.3% of women of both groups (mean age: 43.1 ± 10.6 yrs [Group A], 38.9 ± 8 [Group B] yrs) reported OAB symptoms, 73.3% had stress urinary incontinence and 46.7% reported mixed urinary incontinence. Regarding Group A, 10 patients had CI during penetration, 12 during orgasm and 8 had both forms of CI. The comparison of the responses between Group A and Group B demonstrated a statistically difference (*p* < 0.00). The content validity was assessed by a panel of expert functional urologists. The Cronbach’s alpha coefficients for the total score were high (0.9–1), indicating high internal consistency. The difference between the two groups revealed an internal consistency of IFCI-Q of 0.563–0.851. The test–retest procedure revealed that the k-values of each item are very good.

**Conclusions:**

The Arabic version will allow utilizing this tool in a large population of Arabic-speaking countries, with different ethnic and demographic backgrounds.

## Introduction

Coital urinary incontinence (CI) is an underreported problem which may occur during penetration, orgasm, or both. It has great impact on both sexual and psychological lives of incontinent women [1]. Overall prevalence of CI is about 2%, which may increase to 10–66% in incontinent women [2]. Women with female sexual dysfunction (FSD) and CI rarely report their sexual disturbances spontaneously. It has been reported that only 3% of women complain of FSD, including CI, which may increase to 20% with direct questioning [3]. Female patients with CI have significant deterioration of their quality of life (QoL) and quality of sexual intercourse, which may lead to complete abandonment of their sexual activity [4–6].

To evaluate the impact of CI in clinical practice, we need to evaluate properly the patient’s symptoms using validated questionnaires. Many questionnaires are available to evaluate FSD such as female sexual function index (FSFI) [7] and brief index of sexual functioning for women (BISF-W) [8]. However, none of these evaluate the CI. Recently, a validated questionnaire specific on CI, the International Female Coital Incontinence Questionnaire (IFCI-Q), validated in Italian was published. Gubbiotti M. et al. developed a self-administered questionnaire that evaluates the presence, severity and type of CI and its impact on quality of sexual activity [9]. This questionnaire also investigates the presence of concomitant urinary symptoms.

Re-validation is an important and crucial step when a questionnaire has been translated for use in alternative languages or cultures. This is attributed to the psychometric properties of the original questionnaire which are not always or accurately transferable [10].

The objective of this study is to translate and validate the IFCI-Q Questionnaire in the Arabic (Egyptian) language to provide a reliable evaluation tool for use in Arabic-speaking countries, where many patients do not speak or read other languages. This will enable urologists and urogynecologists to clearly evaluate the CI using a symptom-specific validated tool. This translated version of the questionnaire will be more applicable in Arab-speaking countries because most females feel embarrassed discussing this issue.

## Materials and methods

Local institutional review board and ethics committee approvals were obtained. The Arabic (Egyptian) linguistic translation of the IFCI-Q questionnaire was performed via a multi-step process following the guidelines for cross-cultural adaptation of health-related QoL measures [11]. The IFCI-Q questionnaire was translated from Italian to Arabic language simultaneously and separately by two Egyptian native speaker urologists. Simple Arabic (Egyptian) language was used to be understood by all people, including those with lower socioeconomic/educational levels. Following review and comparison of these two versions, the final Arabic version was developed, and then back-translated into Italian by a professional translator, who was not medical staff and was not familiar with the questionnaire, whose Arabic is the native tongue language, and Italian is his first foreign language. This was followed by back-translation to Italian from Arabic version by another Egyptian native speaker urologist for simplicity, comprehensibility, and accuracy of the content. The original version of the IFCI-Q and the back-translated version were compared, and differences were resolved in another meeting. A small pilot study was performed on five females who complain of CI. This step was to make sure that the questionnaire is vivid and understandable (Appendix). All participants said that the language was simple and not difficult to comprehend.

Two groups were included in the study. The first group (Group A) comprised female patients with almost three months history of CI. The second group (Group B) comprised sexually active females without CI, who attended the urologic clinic for other complaints, such as upper urinary tract conditions, non-infectious diseases of the lower urinary tract or non-oncological pathological conditions, and not due to lower urinary tract symptoms (LUTS). We also recorded participants’ demographic characteristics. Inclusion criteria were adults over 18 years, sexually active, able to read and write in Arabic, and without cognitive disorders. Exclusion Criteria were the inability to read, write and understand the questionnaire, psychiatric and neurologic diseases, cognitive impairment and medications intake such as anticholinergics or alpha blockers”.

Test–retest reliability was determined by administering the questionnaire twice to all females with CI included in the study, with a two-week test–retest time interval between the first and second administration.

## Statistical analysis

The power of the study was calculated with the G*Power program (University of Dusseldorf, Germany), applying an effect size of 0.6 for the two-tailed t-test and 0.05 for alpha error protection.

Considering the main outcomes of study, the sample size was calculated as 30 cases for each group, utilizing one proportion formula, and assumptions of 95% confidence, 80% power, and accepted difference of 60% in effect size. The content validity was assessed by a panel of expert functional urologists. The reliability of the Arabic IFCI-Q was evaluated for internal consistency using Cronbach alpha coefficient for each domain. A Cronbach’s α value of ≥0.70 was considered acceptable for internal consistency for Likert scale variables. Test–retest reliability was determined using the Weighted Cohen’s k-test (k-value ranging from 0 to 1). The discrimination validity was evaluated by comparing the scores of patients with those of the healthy females not complaining of CI using the Mann–Whitney test. In all tests, *P* < 0.05 was considered statistically significant.

## Results

Thirty women complaining of CI (Group A), and 30 women without CI (Group B-Control) completed the final version of the IFCI-Q. The recruitment period started on the 1st June 2022 and finished on the 30st September 2022. Patients of Group A filled the questionnaire twice, with two weeks interval in between for the assessment of test–retest reliability. Mean ± SD age of subjects of Group A and Group B were 43.1 ± 10.6 years, and 38.9 ± 8 years respectively. Basic characteristics of the patients (comorbidities, parity and education level) are reported in the Table 1, without showing any statistical difference between the two groups. The vast majority of patients (83.3%) complained of urinary symptoms: 25/30 (83.3%) reported increased daily – urinary frequency, 17/30 (56.6%) had nocturia, 14/30 (46.7%) urgency, 22/30 (73.3%) stress urinary incontinence (SUI) and 14/30 (46.7%) mixed urinary incontinence (MUI).

**Table 1.** Basic characteristics of the patients.

	Group A (n.pts/%)	Group B (n.pts/%)	p
**Comorbidities**			
	1/30 DM (3.3%) 5/30 HTN (16.6%)	1/30 DM (3.3%) 3/30 HTN (10%)	.8
**Parity**			
	3.2 ± .8	3.1 ± 1	1
**Educational status**			
None	9/30 (30%)	8/30 (26.6%)	1
Elementary	5/30 (16.7%)	7/30 (23.3%)	.9
Preparatory	3/30 (10%)	2/30 (6.6%)	1
High school	8/30 (26.6%)	7/30 (23.3%)	1
University	5/30 (16.7%)	6/30 (20%)	1

DM: diabetes mellitus; HTN: systemic arterial hypertension.

*p* < 0.05 was considered statistically significant.

Regarding the type of CI, 10 patients (33.3%) had CI during penetration, 12 (40%) patients complained of CI during orgasm, and 8 (26.7%) patients had the combined form of CI (during penetration and orgasm). Among women referring CI during penetration, 8/10 (80%) reported SUI, while all the women with CI during orgasm had concomitant OAB symptoms. Only 1/30 patients claimed not to be depressed due to the presence of CI, while 29/30 (96.6%) women stated that they were depressed (sometimes: 8/29 (27.6%) patients; most of the times: 10/29 (34.5%); always: 11/29 (37.9%)).

The comparison of the responses of the patients between the two groups is shown in Table 2, demonstrating a statistically significant difference. Results of the responses of IFCI-Q test and re test are shown in Table 3. There were no statistically significant differences between Group A and Group B, as regards the urinary symptoms.

**Table 2.** Comparison of all questions between Group A and Group B.

Questions	Group A (mean ± SD)	Group B (mean ± SD)	p
**1** (range: 0–1)	1 ± 0	0	.00
**2** (range: 1–2)	1.36 ± .5	–	–
**3** (range: 0–1)	.8 ± .4	–	–
**4** (range: 0–3)	1.8 ± .8	–	–
**5** (range: 0–1)	1 ± 0	.8 ± .4	.00
**6** (range: 0–1)	.8 ± .4	0	.00
**7** (range: 0–3)	2 ± .9	0	.00
**8** (range: 0–1)	.5 ± .5	0	.00

**Table 3.** Results of IFCI-Q test and retest.

Questions	Group A PRE (mean ± SD)	Group A POST (mean ± SD)
**1** (range: 0–1)	1 ± 0	1 ± 0
**2** (range: 1–2)	1.3 ± .5	1.3 ± .4
**3** (range: 0–1)	.8 ± .4	.8 ± .9
**4** (range: 0–3)	1.8 ± .8	1.8 ± .7
**5** (range: 0–1)	1 ± 0	1 ± 0
**6** (range: 0–1)	.8 ± .4	.8 ± .4
**7** (range: 0–3)	2 ± .9	1.9 ± .9
**8** (range: 0–1)	.5 ± .5	.4 ± .5

### Validity

We interviewed 30 women with different ages (range: 27–63 years old) and occupations (housewife, teacher, nurse, or physician), who had completed the IFCI-Q. All of them stated that the questionnaire was easy to understand and to complete, without any difficulty reported. Compile time is between 10 and 12 minutes, in accordance with the original Italian questionnaire.

### Reliability

Reliability evaluates the consistency and replicability of data. Consistency is measured through a formal indicator, the ‘reliability coefficient’ [12]. Internal consistency was in the acceptable range. Thirty women completed the Arabic version of IFCI-Q, twice with a 14-day interval. The Cronbach’s alpha coefficients for the total score were high: Cronbach α = 0.737; 95% CI: 0.571–0.853. The 8 questions have a high correlation. Test–retest (Table 2) showed k-values for each question to be very good, as shown in Table 4.

**Table 4.** The k-values calculated for each question of the questionnaire.

Item	Cohen’s k	95% CI
1	1.000	1.000–1.000
2	1.000	1.000–1.000
3	1.000	1.000–1.000
4	0.912	0.819–1.000
5	1.000	1.000–1.000
5.1	0.958	0.906–1.000
6	0.941	0.800–1.000
6.1	0.953	0.889–1.000
7	0.922	0.822–1.000
8	0.928	0.803–1.000

CI: coital incontinence.

## Discussion

The IFCI-Q is a questionnaire which specifically aims to assess CI. The other tools used to evaluate sexual disorders do not include specific domains on this condition (i.e. Golombok- Rust Inventory of Sexual Satisfaction [GRISS], Pelvic Organ Prolapse/Urinary Incontinence Sexual Questionnaire [PISQ], Brief Index of Sexual Function for Women [BISF-W], Female Sexual Function Index [FSFI]) [13]. In this study, Arabic (Egyptian) linguistic translation of the original questionnaire was performed to allow its use in a large population of Arab-speaking countries. Main characteristics of this tool are the capability to ensure the occurrence and prevalence of CI with clear and direct questions, and to differentiate between the types of CI. The questionnaire also investigates the severity of CI and its relationship with LUTS. Moreover, the emotional condition of these women is also explored with two questions on the QoL and depression.

Validity and reliability of the questionnaire were also confirmed, and data were comparable to those reported in the original Italian version [9]. The majority of the women reported LUTS, and OAB symptoms were very common. Interestingly, the Egyptian sample reported a high prevalence rate of SUI and MUI, while in the Italian population, these symptoms were less. This is the only relevant difference between these two studies.

CI during orgasm was always related to OAB symptoms, while CI during penetration was highly correlated to SUI. These findings were comparable to the previous results of the Italian paper and confirmed thorough the use of a specific standardized tool, the most recognised pathophysiological mechanism of CI [14]. The proposed theory for pathophysiology of CI assumes that females with OAB symptoms due to detrusor overactivity tend to have CI at orgasm, while women with SUI tend to have CI during penetration [14]. This data was not confirmed by some studies, and a possible explanation could be related to the lack of a standardized tool or a questionnaire for CI [15,16]. To date, the available CI results have been reported from studies that have investigated this condition through specific questions or through the improper use of parts of other questionnaires, but not in a standardized validated way.

Validated questionnaires are commonly used to better evaluate pathological disorders, collecting more reliable, reproducible, and standardized data. Both the current study and the Italian one have used the same validated standardized and specific questionnaire (IFCI-Q), and they have found the same type of CI (during orgasm) related to OAB symptoms, and the same kind of CI (during penetration) attributed to SUI. Among the results of these studies, a high reproducibility and correspondence on CI and symptoms were found by means of the use of IFCI-Q. Present data support the main pathophysiological theory on of CI proposed by Hilton [14]. The strength of validation of the Arabic- language IFCI-Q is to allow more appropriate assessment and diagnosis of CI in a large cohort of population. Additional data obtained thorough this standardized tool might help better and more accurate understanding of this condition.

Interestingly, this study confirmed the negative influence of CI on the emotional condition, with almost all the women reporting depression. The negative impact of OAB on female sexuality is well known and proved, and therefore it is not surprising that women with a urinary disorder during intercourse had a compromised emotional state as well [17,18].

Limitation of this study is the small sample size. However, statistical power was reached, and it is challenging to find women ready to talk about this embarrassing condition. This questionnaire may represent an easier way to understand the magnitude of this problem and talk about it more easily with females.

## Conclusions

IFCI-Q is a validated questionnaire investigating CI, allowing to collect more reliable, reproducible, and standardized data. The Arabic (Egyptian) version will allow utilizing this tool in a large population of Arabic-speaking countries, with different ethnic and demographic backgrounds.
